# Structural analysis and corrosion studies on an ISO 5832-9 biomedical alloy with TiO_2_ sol–gel layers

**DOI:** 10.1007/s10856-013-5099-7

**Published:** 2013-11-23

**Authors:** B. Burnat, G. Dercz, T. Blaszczyk

**Affiliations:** 1 Department of Inorganic and Analytical Chemistry, Faculty of Chemistry, University of Lodz, Tamka 12, 91-403 Lodz, Poland; 2 Institute of Materials Science, University of Silesia, 75 Pulku Piechoty 1A, 41-500 Chorzow, Poland

## Abstract

The aim of this study was to demonstrate the relationship between the structural and corrosion properties of an ISO 5832-9 biomedical alloy modified with titanium dioxide (TiO_2_) layers. These layers were obtained via the sol–gel method by acid-catalyzed hydrolysis of titanium isopropoxide in isopropanol solution. To obtain TiO_2_ layers with different structural properties, the coated samples were annealed at temperatures of 200, 300, 400, 450, 500, 600 and 800 °C for 2 h. For all the prepared samples, accelerated corrosion measurements were performed in Tyrode’s physiological solution using electrochemical methods. The most important corrosion parameters were determined: corrosion potential, polarization resistance, corrosion rate, breakdown and repassivation potentials. Corrosion damage was analyzed using scanning electron microscopy. Structural analysis was carried out for selected TiO_2_ coatings annealed at 200, 400, 600 and 800 °C. In addition, the morphology, chemical composition, crystallinity, thickness and density of the deposited TiO_2_ layers were determined using suitable electron and X-ray measurement methods. It was shown that the structure and character of interactions between substrate and deposited TiO_2_ layers depended on annealing temperature. All the obtained TiO_2_ coatings exhibit anticorrosion properties, but these properties are related to the crystalline structure and character of substrate–layer interaction. From the point of view of corrosion, the best TiO_2_ sol–gel coatings for stainless steel intended for biomedical applications seem to be those obtained at 400 °C.

## Introduction

Biomaterials belong to the group of modern materials which are used for the repair and reconstruction of human body. Some of the application examples of those materials are prostheses and implants that are used in nearly every medical discipline. A very important group of biomaterials are metallic materials—pure metals and alloys. FeCrNi alloys are the most often used biomaterials for bone implants and surgical instruments; for example, the popular AISI 316L (ISO 5832-1) stainless steel and the comparable Rex 734 (ISO 5832-9) alloy. The good mechanical properties and biocompatibility of both the mentioned FeCrNi alloys make them very attractive for biomedical applications [1]. However, it is known that steel implants corrode in the body. They release iron, chromium and nickel ions, which can cause severe allergic reactions [2]. Rex 734 is quite a new alloy, which is more corrosion resistant than AISI 316L steel [3]. The chemical composition of both the mentioned alloys according to ISO standards is presented in Table 1 [4].

**Table 1:** Chemical composition of AISI 316L stainless steel and Rex 734 alloy (wt%)

Element	C	Si	Mn	P	S	Cr	Ni	Mo	Cu	N	Nb	Fe
AISI 316L (ISO 5832-1)	Max. 0.030	Max. 1.0	Max. 2.0	Max. 0.025	Max. 0.010	17.0−19.0	13.0−15.0	2.25−3.5	Max. 0.50	Max. 0.10	–	Rest
Rex 734 (ISO 5832-9)	Max. 0.08	Max. 0.75	2.00−4.25	Max. 0.025	Max. 0.01	19.5−22.0	9.0−11.0	2.0−3.0	Max. 0.25	0.25−0.50	0.25−0.80	Rest

Various surface modification techniques have been developed to improve the properties of metallic biomaterials, especially their corrosion resistance and biocompatibility. One of the most effective methods is to cover the metal surface with a ceramic coating, e.g., nitrides, carbides and transition metal oxides. Coatings on metal surfaces can be created in different ways: the PVD [5] and CVD [6] methods from gaseous phase or the sol–gel method [7] and electrodeposition [8] from the liquid phase. The sol–gel method is often used for deposition of oxide films such as TiO_2_, SiO_2_ and Al_2_O_3_ [9–13]. They are used as single-layer or multi-layer coatings. Sol–gel coatings show excellent chemical stability, oxidation control and provide enhanced corrosion resistance for metal substrates [13]. This deposition technique offers various advantages, including the precise control of the chemical composition, thickness, and microstructure of the coating and the possibility to prepare homogeneous films [14]. The sol–gel method enables the production of amorphous or crystalline layers depending on the applied temperature [10]. In comparison with the other coating methods, the sol–gel process requires less equipment and so is less expensive.

Titanium dioxide (TiO_2_, titania) is an important ceramic material with versatile applications due to its self-cleaning character, biocompatibility and corrosion resistance. Some positive properties of titanium dioxide depend on its phase structure. TiO_2_ exists in three different crystal phases: rutile, anatase and brookite. Rutile is a thermodynamic stable state, while the other two phases are metastable states [15]. Anatase is the low-temperature form of TiO_2_ (300–550 °C) and it transforms into rutile during heating (ca. 1100 °C) [16]. Other authors report that the transformation from anatase to rutile proceeds in the temperature range of 500–750 °C and both TiO_2_ forms exist in films. Above the temperature of 750 °C, TiO_2_ exists as pure rutile [10].

The literature describes the different properties of TiO_2_ coatings, deposited on various substrates including silicon wafers, glasses and metallic materials (e.g. steels). Most of the publications about TiO_2_ films describe their phase structure, thickness, porosity and adherence to substrates. The influence of TiO_2_ coatings on the corrosion properties of stainless steel are also reported in the literature [17–23]. Several authors stated that TiO_2_ films improved corrosion resistance by acting as a protective barrier on the steel surface [18–20, 23–25].

There are only a few reports describing the nature of the steel substrate—TiO_2_ film connection [26, 27]. Evans [26] wrote that the components of stainless steel (mainly Fe and/or Cr) diffuse into the titania film. Zhu et al. [27] reported that Fe diffuses from the steel substrate into the TiO_2_ film and reacts with O_2_ from the air. As a result, an interlayer of iron oxide forms during the annealing process. Zhu et al. [27] stated that Fe in the TiO_2_ layer and interlayer exists as a rhombohedral Fe_2_O_3_ species. The author asserts as well that the degree of diffusion is associated with increasing annealing temperature and time. Such diffusion has a deleterious effect on the photoactivity of the titania films obtained by CVD or sol–gel methods [26, 27]. Unfortunately, in the literature there is no information about the influence of the mentioned interlayers on the corrosion properties of steel coated by TiO_2_ sol–gel layers.

The objective of this study was to demonstrate the relationship between the structural and corrosion properties of an ISO 5832-9 biomedical alloy modified with TiO_2_ sol–gel layers. Different structural properties of TiO_2_ layers were obtained by applying a variety of annealing temperatures. The morphology, chemical composition, crystallinity, thickness and density of TiO_2_ layers were characterized by X-ray measurement methods. The interaction type and boundary structure of the substrate-TiO_2_ layer were determined on the basis of structural analysis results. The corrosion behavior of the examined alloy coated by TiO_2_ was determined in Tyrode’s physiological solution using electrochemical methods. Several corrosion parameters were determined: corrosion potential, polarization resistance, corrosion rate (CR), breakdown and repassivation potentials. Corrosion damage was analyzed using scanning electron microscopy.

## Experimental

### Materials

The biomedical alloy Rex 734 (MEDGAL, Białystok, Poland) was used as a metallic substrate. Its chemical composition is as follows (wt%): Cr (20.79), Ni (9.81), Mo (2.22), Mn (4.07), Nb (0.32), N (0.40), Si (0.40), Cu (0.07), C (0.034), S (≤0.002), P (0.019), Al + Co + V (0.19) and Fe (balance). Rex 734 alloy samples were discs with a diameter of 28 mm and height ca. 3 mm. Sample surfaces were grinded on SiC abrasive paper, mechanically polished with Al_2_O_3_ suspension and cleaned in an ultrasonic bath. The last stage of the surface preparation procedure included etching of sample surfaces for a short time in a mixture of 2 % HF, 10 % HNO_3_ and 88 % H_2_O. Next, the samples were cleaned in an ultrasonic bath again, rinsed with ethanol and dried with Ar (99.999 %). Samples prepared in such a way were ready for TiO_2_ coating.

TiO_2_ layers were obtained from sol, in which titanium (IV) isopropoxide Ti[OCH(CH_3_)_2_]_4_ (97 %, Aldrich) was used as a precursor, isopropanol (99.7 %, POCh) as a medium and 2 M HCl (POCh) as a catalyst. Such sol was used by Piwoński for synthesis of non-porous TiO_2_ layers [28].

All the chemical reagents used in the experiment were analytical grade and were applied without further purification.

### Synthesis of TiO_2_

The preparation of TiO_2_ sol solution was started by mixing 0.00422 mol (1.2 g) of titanium (IV) isopropoxide with 0.22 mol (13.2 g) of isopropanol. Next, 0.0011 mol (0.04 g) of hydrochloric acid was added dropwise to the solution and then the mixture was stirred vigorously for 45 min at room temperature. The resultant titanium precursor sol was homogenous and clear. It could be used for the preparation of TiO_2_ films on Rex 734 alloy samples.

The TiO_2_ layers were obtained by immersing the sample once into the sol using DCMono 75 (NIMA Technology). The immersion speed and withdrawal speed were 20 mm min^−1^ (0.33 mm s^−1^) and the immersion time in bottom state in sol was 30 s. Even during withdrawal of the already coated sample, the solvent evaporated and at the same time the alkoxide was hydrolyzed by the atmospheric water, forming titanium hydroxide and isopropanol according to reaction (1):

1 $${\text{Ti}}\left[ {{\text{OCH(CH}}_{3} )_{2} } \right]_{4} + 4{\text{H}}_{ 2} {\text{O}}{\mathop{\longrightarrow}\limits^{{{\text{H}}^{ + } }}}{\text{Ti}}\left( {\text{OH}} \right)_{4} + 4\left( {{\text{CH}}_{ 3} } \right)_{2} {\text{CHOH}}$$

During ongoing acid-catalyzed hydrolysis, polycondensation of the hydrolyzed particles occurred. Ti(OH)_4_ molecules formed $- {\text{Ti}} - {\text{O}} - {\text{Ti}} -$ connections by elimination of water according to reaction (2):

2 $$2 {\text{Ti(OH)}}_{ 4} \to ( {\text{OH)}}_{ 3} {\text{Ti}} - {\text{O}} - {\text{Ti(OH)}}_{ 3} + {\text{H}}_{ 2} {\text{O}}$$

A three-dimensional network of $- {\text{Ti}} - {\text{O}} - {\text{Ti}} -$ connections formed, when reaction (2) was repeated. As a result, amorphous TiO_2_ particles were formed.

After the coating process, the samples were initially dried at 100 °C for 2 h in the air using a muffle furnace. During drying, most of the isopropanol and water evaporated and an agglomeration of the TiO_2_ particles was created in the film (i.e. gel formation). Next, the samples were divided into seven groups and each group was annealed at different temperatures: 200, 300, 400, 450, 500, 600, 800 °C for 2 h. The rate of temperature increase was 10 °C min^−1^. Finally, the samples were cooled to room temperature in the furnace. The resultant TiO_2_ layers assumed different colors, depending on the annealing temperature. For example, after heating at 200 and 400 °C the layers were gold, after heat treatment at 600 °C they were blue, while at 800 °C they became grey. At all temperatures, the resultant TiO_2_ layers were homogeneous over their whole surface and were free of cracks.

### Characterization of TiO_2_ layers

Structural analysis was carried out for selected TiO_2_ coatings annealed at 200, 400, 600 and 800 °C.

All X-ray diffraction experiments were carried out using a X-Pert Philips PW 3040/60 diffractometer, operated at 30 mA and 40 kV, equipped with a vertical goniometer and an Eulerian cradle. The length of radiation (λCu_Kα_) was 1.54178 Å.

The set-ups of the GIXD (Grazing Incident X-ray diffraction) and XRR (X-Ray reflectivity) optics are detailed in Table 2.

**Table 2:** X-ray beam formation for asymmetrical and GIXD, XRR geometry

Geometry	Soller slit (rad)	Div. slit (°)	Mask width (mm)	Anti-scatter slit (°)	Rec. slit (mm)	Soller slit (rad)	Curved crystal monochromator
GIXD	0.04	1/32	5	1/32	0.3	0.04	PW3123/10
XRR	0.04	1/32	5	1/32	0.3	0.04	PW3123/10

The GIXD diffraction patterns were registered in 2*θ* range from 10° to 120° and 0.05° step for the incident angle *α*: 0.25; 0.50; 1.00; 2.50 and 5.00 degrees, respectively. In order to maintain comparable intensities of the diffraction lines, the conditions for collecting patterns (step and counting time) were properly adjusted. GIXD uses small incident angles (*α*) for the incident X-ray beam, so that it is used to study surface layers as the beam penetration is limited. Distances are in the order of nanometers. The *α* angle of incidence is fixed, so that the degree of penetration by the X-rays into the sample remains constant throughout the measurement. At low *α*-angles of incidence, the X-rays penetrate only the uppermost layers of a sample. At higher *α*-angles of incidence, the X-rays penetrate more deeply into the sample [29, 30].

The reflectometric curves were collected in 2*θ* range from 0.15° to 3° and step 0.005°. An attenuator was used in order to reduce scattered intensity. XRR is based on measuring the scattering from the layer and the substrate that differ in their density. This method provides information about layer thickness and density [31, 32]. The parameters of the layer were found by fitting the experimental reflectometric curves to the theoretical curves [33].

The SEM (JEOL JSM-6480) and EDS techniques were used in the analysis of sample morphology and its chemical composition, respectively.

### Corrosion measurements

The corrosion properties of Rex 734 alloy samples with and without TiO_2_ sol–gel layers were determined from accelerated corrosion measurements. The measurements were carried out in Tyrode’s physiological solution at a temperature of 37 °C using potentiostat/galvanostat PGSTAT 30/1 (EcoChemie Autolab). The chemical composition of used corrosion solution is presented in Table 3.

**Table 3:** Chemical composition of Tyrode’s solution

	NaCl	KCl	CaCl_2_	NaHCO_3_	MgCl_2_·6H_2_O	NaH_2_PO_4_·H_2_O
[g dm^−3^]	8.000	0.200	0.200	1.000	2.135	0.0575

Electrochemical measurements were carried out in a three-electrode electrolytic cell consisting of a saturated calomel electrode as reference electrode, a platinum foil as counter electrode, and a sample (the exposed area was 0.64 cm^2^) as working electrode. All potentials presented in the paper are given versus used saturated calomel electrode (E° = 0.236 V vs. standard hydrogen electrode).

The corrosion solution was deareated with pure argon for 15 min both before and also during measurement. Accelerated corrosion measurements were carried out according to the following measurement cycle:

1. The measurement of corrosion potential *E*_*cor*_ in an open circuit (OCP);
2. The measurement of polarization resistance *R*_*p*_ according to Stern–Geary’s method in a scanning range ±20 mV versus *E*_*cor*_ potential with a scan rate of 0.5 mV s^−1^;
3. The measurement of potentiodynamic characteristic from 0.2 V below the corrosion potential (previously stabilized) towards the anodic direction with a scan rate of 1.0 mV s^−1^. When the current density reached 5 mA cm^−2^, the potential sweep was reversed and the backward branch was registered up to the starting potential.

The number of samples used for every corrosion test was three. Results presented in this paper are averaged values.

## Results

### Phase analysis of TiO_2_ layers

Diffraction patterns of Rex 734 alloy samples with TiO_2_ layers, annealed at 200, 400, 600 and 800 °C, were registered by means of the GIXD method with incidence angles α: 0.25; 0.50; 1.00; 2.50 and 5.00 degrees.

**Fig. 1:**
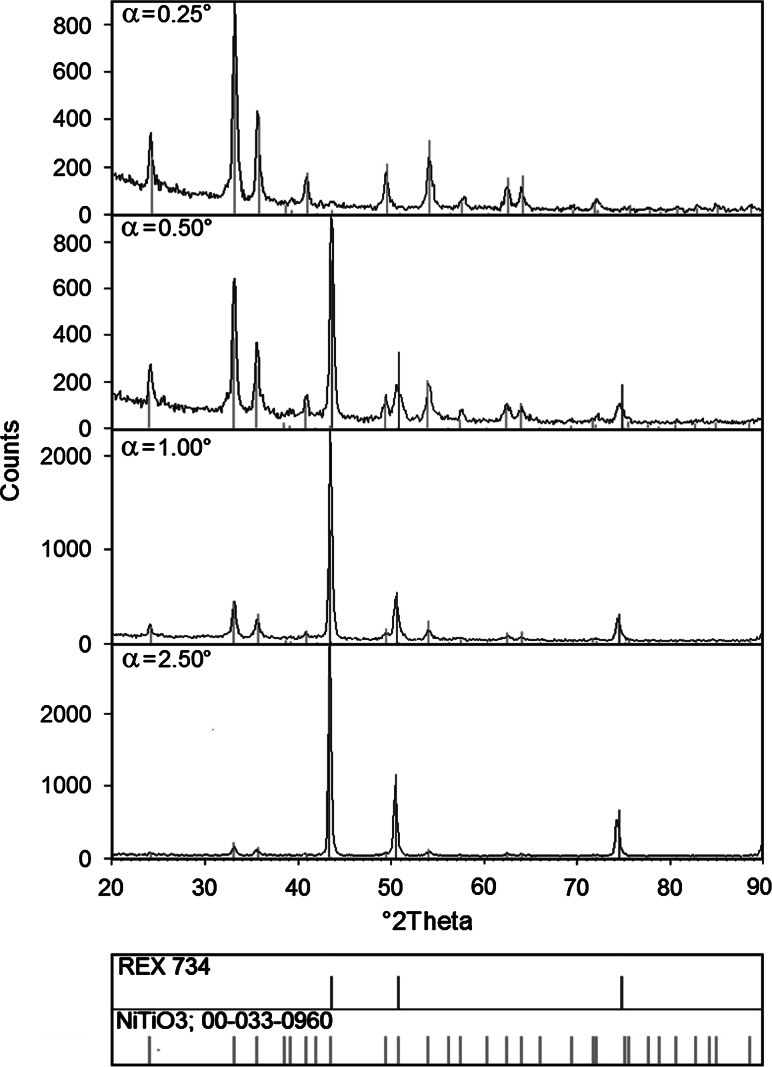
Diffraction pattern and phase analysis of Rex 734 alloy sample with TiO_2_ layer annealed at 600 °C

**Fig. 2:**
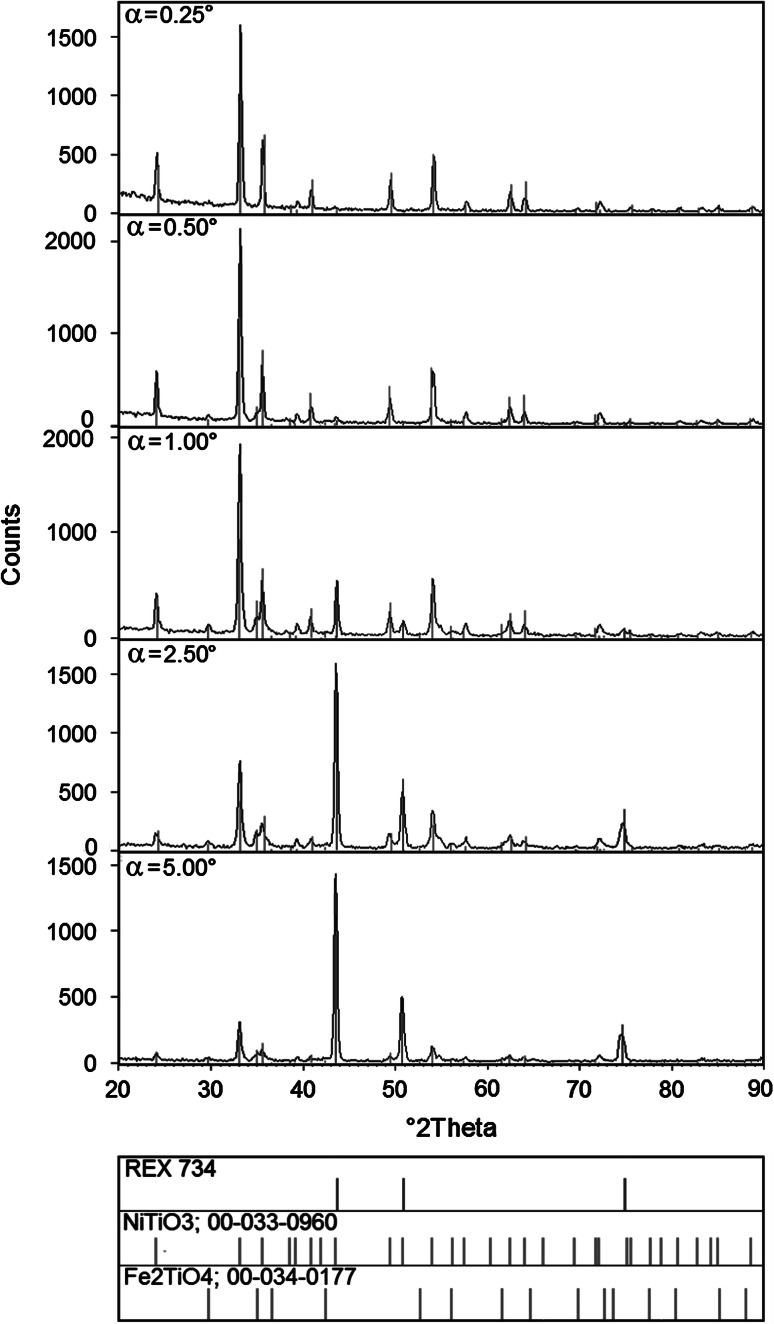
Diffraction pattern and phase analysis of the Rex 734 alloy sample with TiO_2_ layer annealed at 800 °C

The diffraction patterns (*α* = 0.25 and *α* = 0.50) of the Rex 734 alloy samples with TiO_2_ layers annealed at 200 and 400 °C show that the titania layer has an amorphous character. Annealing at higher temperatures, i.e. 600 and 800 °C, results in the crystallization of the layer and the formation of new TiO_2_-based phases because of diffusion of the substrate elements. Qualitative phase analysis of samples with TiO_2_ annealed at 600 °C shows that the layer is composed of NiTiO_3_ (ICDD PDF 33-0960) phase (Fig. 1). In the case of samples annealed at 800 °C, NiTiO_3_ (ICDD PDF 33-0960) and Fe_2_TiO_4_ (ICDD PDF 34-0177) phases are observed (Fig. 2). Moreover, in the case of annealing at the last temperature, a Fe_2_O_3_ (ICDD PDF 85-0987) phase can also be detected. Difficulties in matching Fe_2_O_3_ phase are the result of strong overlapping of the diffraction lines from NiTiO_3_ and Fe_2_O_3_ phases. Catalog positions of 2*θ* for the both phases (NiTiO_3_ and Fe_2_O_3_) overlap and the only reflection that could be matched to the Fe_2_O_3_ phase is the one at 2*θ* of 39.42. The identification of the iron oxide is in accordance with the chemical EDS analysis of the big, “protruding”, porous grains containing oxide and iron (Table 8). It should be noted that the Fe_2_TiO_4_ phase emerges when the incidence angle is *α* = 0.50° and that the greater the incidence angle *α*, the more intense the diffraction lines and there is also a simultaneous decrease in the intensity of reflections from the NiTiO_3_ phase. These changes confirm that only NiTiO_3_ is present on the surface of the layer. Furthermore, there is a continuous change of the phase content inside. Reflections that come from the Rex 734 substrate are visible for all samples.

### Thickness and density of TiO_2_ layers

**Fig. 3:**
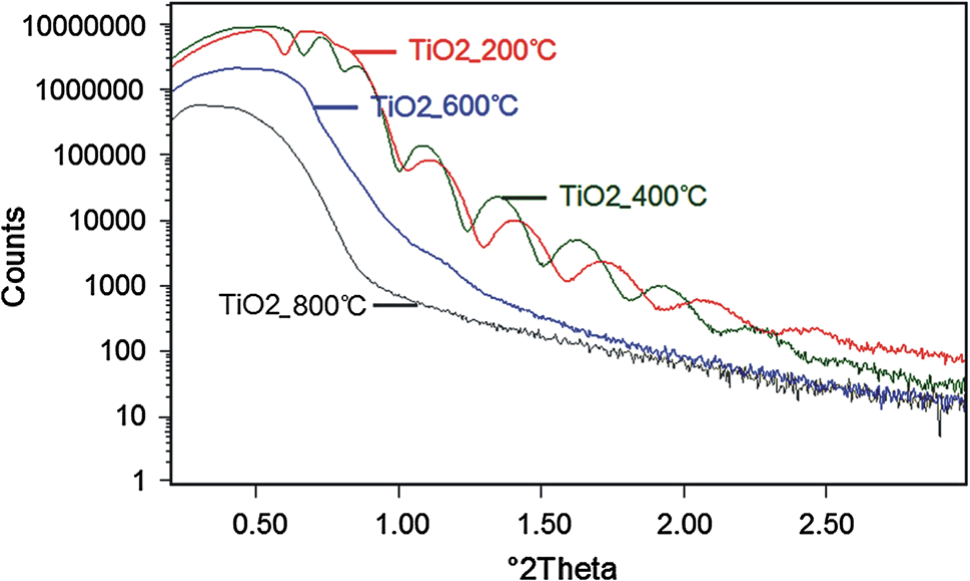
Reflectometric curves collected for Rex 734 alloy with TiO_2_ layers annealed at: 200, 400, 600 and 800 °C

The thickness and density *ρ* of the deposited TiO_2_ layers were determined using the reflectometry technique with the WinGix program. The reflectometric curves obtained for the Rex 734 alloy with TiO_2_ layers annealed at 200, 400, 600 and 800 °C, have different shapes (Fig. 3), which indicates the differences in the layer’s construction for individual samples.

Classic reflectometric curves were obtained only for Rex 734 samples with TiO_2_ layers annealed at 200 and 400 °C. Therefore, it was possible to determine the value of the critical *γ* angle for these samples. The thickness of these layers determined on the basis of the fluctuation intensity equals 266 Å for 200 °C and 295 Å for 400 °C. In the case of samples with TiO_2_ annealed at 600 and 800 °C the reflectometric curves obtained are typical for layers of infinite thickness.

The value of the density of TiO_2_ layers annealed at 200 °C (*ρ* = 3.91 g cm^−3^) confirms that the amorphous layer contains titania in the form of anatase (*ρ* = 3.89 g cm^−3^). The higher density of the TiO_2_ layer annealed at 400 °C (*ρ* = 4.43 g cm^−3^) may indicate the intensification of the crystallization process. Annealing of the samples at 600 °C causes subsequent densification of the TiO_2_ layer (*ρ* = 5.07 g cm^−3^). Such a density value is in close correspondence with the catalog density of the NiTiO_3_ phase (*ρ* = 5.10 g cm^−3^). In the case of the layer annealed at 800 °C, a decrease in density to *ρ* = 4.67 g cm^−3^ is observed. The lower density value may be the result of the vacancies formation and the additional Fe_2_TiO_4_ phase (*ρ* = 4.78 g cm^−3^) on the basis of diffusion of alloying elements to the TiO_2_ coating.

The values obtained for thickness and density of TiO_2_ layers are collected in Table 4.

**Table 4:** Values of density and thickness of TiO_2_ layers determined from reflectometric curves

	200 °C	400 °C	600 °C	800 °C
Thickness (Å)	266	295	–	–
*ρ* (g cm^−3^)	3.91	4.43	5.07	4.67

### Corrosion potential

In order to determine the corrosion potential value *E*_*cor*_ for each sample in Tyrode’s solution, the potential-time dependence in relation to a reference electrode was recorded. A stable potential value was reached typically after about 2,000 s. Obtained corrosion potential values are presented in Table 5 with a calculated standard deviation. This table also contains the shift of corrosion potential *ΔE*_*cor*_, as defined by the formula (3):

**Table 5:** Values of corrosion potential *E*_*cor*_ and shift of this potential *ΔE*_*cor*_ for Rex 734 alloy with TiO_2_ layers in Tyrode’s solution

Sample	*E*_*cor*_ (V)	*ΔE*_*cor*_ (V)
Uncoated	−0.283 ± 0.003	0.000
+TiO_2_ 200	−0.054 ± 0.083	0.229
+TiO_2_ 300	0.000 ± 0.035	0.283
+TiO_2_ 400	0.014 ± 0.028	0.297
+TiO_2_ 450	0.121 ± 0.029	0.404
+TiO_2_ 500	0.118 ± 0.029	0.401
+TiO_2_ 600	0.035 ± 0.110	0.318
+TiO_2_ 800	0.080 ± 0.044	0.363

3 $$\varDelta E_{cor} = E_{{corTiO_{2} }} - E_{cor0}$$

where $E_{cor0}$—corrosion potential of an uncoated sample, $E_{{corTiO_{2} }}$—corrosion potential of a sample with TiO_2_ layers.

Analyzing the values of *E*_*cor*_ and *ΔE*_*cor*_, it can be seen that the TiO_2_ layer formed on the Rex 734 alloy surface and annealed at any of the tested temperatures causes an increase in the value of the corrosion potential. This influence depends on the annealing temperature. The smallest increase in the corrosion potential is observed for samples with a layer annealed at 200 °C, and the highest for samples with a layer annealed at temperatures of 450 and 500 °C. For other annealing temperatures, the shift of corrosion potential has intermediate values. Extremely high value for the standard deviation of corrosion potential is observed for samples with TiO_2_ layer annealed at 600 °C. This fact indicates that the surface properties obtained with such modification of the alloy are not repeatable.

### Polarization resistance, corrosion current and corrosion rate

The values of polarization resistance *R*_*p*_ and corrosion current *i*_*cor*_ were calculated from the slope of the Stern-Geary’s characteristics using CorrView software (Scribner Associates Inc.). Using the determined values of polarization resistance, porosity *p*, which is associated with the formation of the TiO_2_ layer, was calculated and this is defined by the following equation [34, 35]:

4 $$p = \frac{{R_{0} }}{{R_{{TiO_{2} }} }}$$

where *R*_0_—polarization resistance of uncoated Rex 734 alloy, $R_{{TiO_{2} }}$—polarization resistance of this alloy with TiO_2_ layers.

*CR* was calculated according to the standard ASTM G 102–89 [36] from the formula:

5 $$CR = K_{1} \frac{{i_{cor} }}{\rho }EW$$

where *K*_*1*_ = 3.27 × 10^−3^ [mm g μA^−1^ cm^−1^ year^−1^], *i*_*cor*_ [μA cm^−2^]—corrosion current; *ρ* [g cm^−3^]—density of alloy. The unit of the *CR* is mm year^−1^.

To calculate the *CR* value, it was assumed that in corrosion potential *E*_*cor*_ only the substrate, Rex 734 alloy, corrodes. No other corrosion processes associated with the formed and annealed TiO_2_ coatings were taken into account in this calculation.

*EW* occurring in the formula for the *CR* is an equivalent weight, which for the alloy was calculated from the following dependence [36]:

6 $$EW = \frac{1}{{\sum {\frac{{n_{i} f_{i} }}{{W_{i} }}} }}$$

where *f*_*i*_ is the mass fraction of the *i*th element in the alloy, *W*_*i*_ is the atomic weight of the *i*th element in the alloy, *n*_*i*_ is the valence of *i*th element of the alloy. According to the ASTM standard [36], only elements above one mass percent in the alloy are included in this calculation. The value of equivalent weight *EW* for the Rex 734 alloy calculated from the above formula equals 19.14.

The determined values for *R*_*p*_, *p*, *i*_*cor*_ and *CR* with standard deviations are listed in Table 6.

**Table 6:** Values of polarization resistance *R*_*p*_, porosity *p*, corrosion current *i*_*cor*_ and corrosion rate *CR* for Rex 734 alloy with TiO_2_ layers in Tyrode’s solution

Sample	*R*_*p*_ (ohm cm^2^)	*p*	*i*_*cor*_ (A cm^−2^)	*CR* (mm year^−1^)
Uncoated	(2.67 ± 0.19) × 10^5^	1.000	(9.81 ± 0.70) × 10^−8^	(7.87 ± 0.57) × 10^−4^
+TiO_2_ 200	(5.60 ± 2.20) × 10^6^	0.048	(7.05 ± 2.70) × 10^−9^	(5.66 ± 2.17) × 10^−5^
+TiO_2_ 300	(8.26 ± 1.04) × 10^6^	0.032	(3.25 ± 0.48) × 10^−9^	(2.61 ± 0.38) × 10^−5^
+TiO_2_ 400	(9.30 ± 0.56) × 10^6^	0.029	(2.84 ± 0.17) × 10^−9^	(2.28 ± 0.14) × 10^−5^
+TiO_2_ 450	(1.28 ± 0.18) × 10^7^	0.021	(2.07 ± 0.30) × 10^−9^	(1.66 ± 0.24) × 10^−5^
+TiO_2_ 500	(7.31 ± 1.28) × 10^6^	0.037	(3.65 ± 0.63) × 10^−9^	(2.93 ± 0.50) × 10^−5^
+TiO_2_ 600	(2.50 ± 1.47) × 10^6^	0.107	(1.72 ± 1.34) × 10^−8^	(1.38 ± 1.08) × 10^−4^
+TiO_2_ 800	(4.19 ± 0.66) × 10^6^	0.064	(6.48 ± 1.17) × 10^−9^	(5.20 ± 0.94) × 10^−5^

As in the case of the corrosion potential, modification of the alloy surface by TiO_2_ layers always increases the polarization resistance. For each annealing temperature, this increase in the *R*_*p*_ value is different: the highest polarization resistance values are shown by samples with a TiO_2_ layer annealed at a temperature of 450 °C, while the lowest values are identified in samples with a layer annealed at 600 °C. In relation to the polarization resistance of uncoated Rex 734 alloy, these values are about 48 times and about 9.4 times higher, respectively. As the corrosion current *i*_*cor*_ and the *CR* are inversely proportional to the polarization resistance *R*_*p*,_ so the changes in *R*_*p*_ values are transferred onto the changes in *i*_*cor*_ and *CR* values. Surface modification with TiO_2_ layers causes a corresponding decrease in the values of both mentioned quantities.

### Potentiodynamic characteristics in the anodic polarization range

**Fig. 4:**
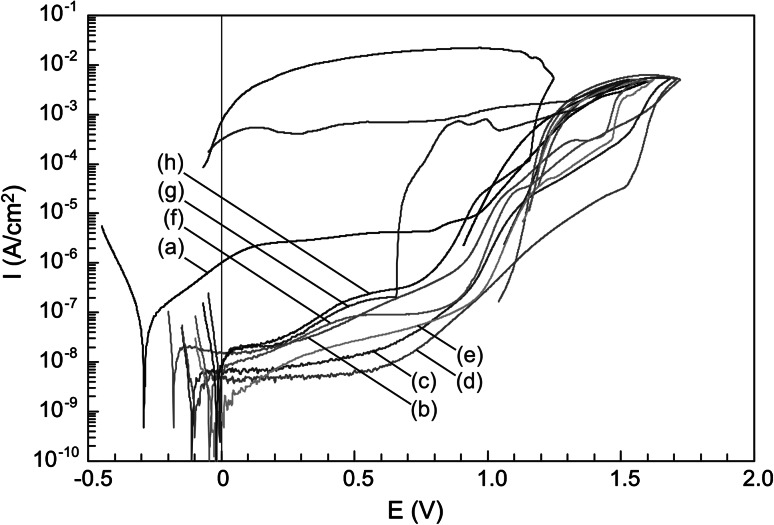
Potentiodynamic characteristics for uncoated Rex 734 alloy (*a*) and for this alloy with TiO_2_ layers annealed at: 200 °C (*b*), 300 °C (*c*), 400 °C (*d*), 450 °C (*e*), 500 °C (*f*), 600 °C (*g*), 800 °C (*h*)

Example potentiodynamic characteristics collected for the Rex 734 alloy (both uncoated and also samples coated with TiO_2_ layers) in Tyrode’s solution are shown in Fig. 4. For greater clarity of the graph, fragments of the return characteristics branches have been truncated.

The shape of potentiodynamic characteristics for all tested samples is typical for localized corrosion. Some corrosion parameters have been determined from characteristics presented in Fig. 4. The values of breakdown *E*_*b*_ and repassivation *E*_*rep*_ potentials and the values of current density *i*_*0.6*_ determined at an arbitrarily chosen polarization potential *E* = 0.6 V [37] are collected in Table 7.

**Table 7:** Selected corrosion parameters as a function of annealing temperature of TiO_2_ layers

Sample	*i*_*0.6*_ (A cm^−2^)	*E*_*b*_ (V)	*E*_*rep*_ (V)
Uncoated	4.14 × 10^−6^	~0.97	0.96
+TiO_2_ 200	1.42 × 10^−7^	~1.38	1.22
+TiO_2_ 300	1.85 × 10^−8^	1.53	1.14
+TiO_2_ 400	8.00 × 10^−9^	1.52	1.10
+TiO_2_ 450	3.51 × 10^−8^	1.47	1.17
+TiO_2_ 500	9.06 × 10^−8^	1.44	1.18
+TiO_2_ 600	1.96 × 10^−7^	0.66	–
+TiO_2_ 800	2.71 × 10^−7^	1.16	–

Analyzing the results, it can be concluded that applying a TiO_2_ sol–gel layer significantly reduces the current density in the passive range. The greatest changes in the current density at a potential of *E* = 0.6 V are observed for the Rex 734 alloy with layers annealed in a temperature range of 300–450 °C—the values of *i*_*0.6*_ for these samples are ca. 100–500 times lower in relation to uncoated samples. Data in Table 7 show that TiO_2_ layers cause changes in the values of breakdown potential. Potentials *E*_*b*_ for uncoated samples and samples with TiO_2_ layer annealed at 200 °C could only be estimated, since there is no sharp increase in current values typical for breakdown processes. For other samples, potential *E*_*b*_ is uniquely determined. The highest values of potential *E*_*b*_, ca. 1.5 V are met in samples with TiO_2_ layers obtained at temperatures in the range of 300–450 °C. However, the lowest value of *E*_*b*_, lower than the uncoated samples, is to be found for samples with a TiO_2_ layer annealed at 600 °C. It is worth noting that in the case of these samples and samples with a TiO_2_ layer annealed at 800 °C, backward branches of potentiodynamic characteristics do not intersect the forward branches. This fact indicates the development of pits formed in the applied potential range. Potentiodynamic characteristics for other Rex 734 alloy samples have a current hysteresis loop associated with repassivation of pits; therefore, the repassivation potential *E*_*rep*_ could be determined. This *E*_*rep*_ potential is relatively low for an uncoated alloy. Higher values of *E*_*rep*_ are seen in samples with TiO_2_ layers annealed at temperatures in the range of 200–500 °C.

### Morphology of TiO_2_ layers

**Fig. 5:**
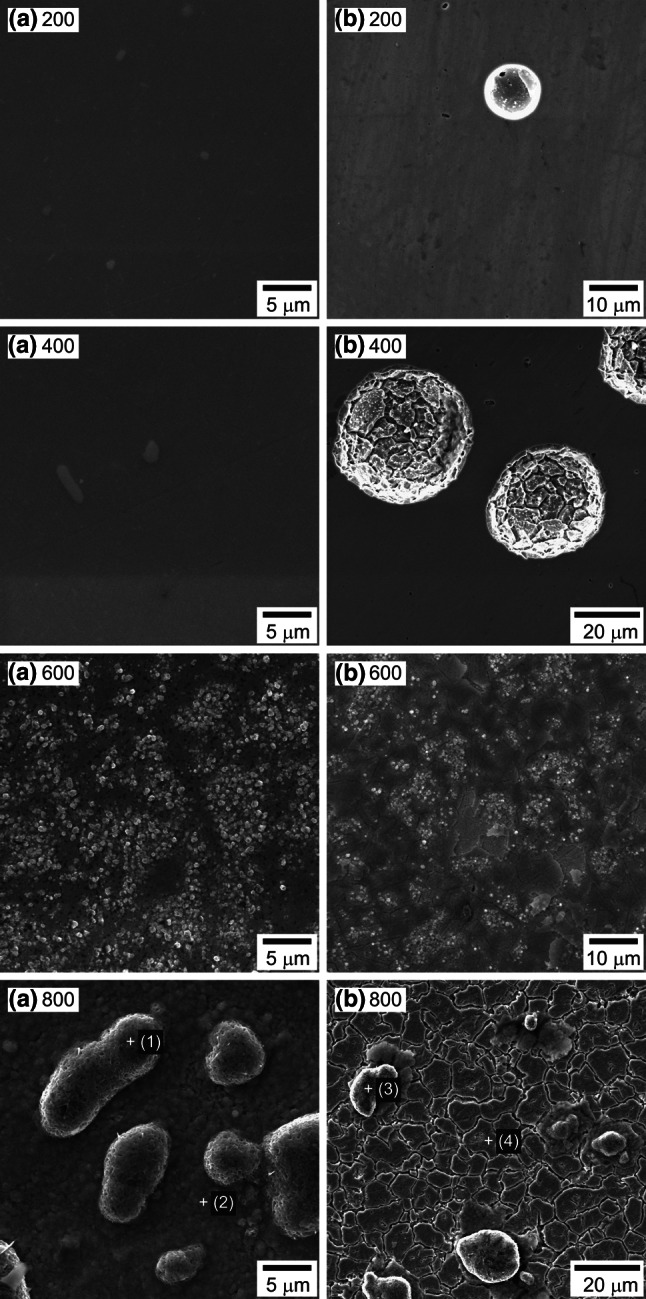
SEM images of the samples with TiO_2_ layers annealed at 200, 400, 600 and 800 °C; **a** as prepared (before corrosion), **b** after anodic polarization in Tyrode’s solution

Surface morphologies of samples with TiO_2_ layers annealed at 200, 400, 600 and 800 °C are shown in Fig. 5. Morphologies marked with (a) refer to the state before corrosion, while those marked with (b) are related to the state after anodic polarization.

SEM analysis of surfaces before anodic polarization shows that randomly located irregularly shaped precipitates occur on the homogeneous surface of samples annealed at 200 and 400 °C. These precipitates have a higher level of Nb, Mo and Cr. Nb levels reach 42 at.% (58 wt%), Mo content is ca. 7.7 at.% (11 wt%), and Cr is ca. 25 at.% (19 wt%). The result of anodic polarization is evident local pitting corrosion manifested by creation of circular pits, stochastically distributed on the surface of samples. SEM analysis conducted after anodic polarization does not show the above mentioned precipitates on the sample surfaces—the elemental composition inside and outside the pits is similar to the original elemental composition of the investigated alloy.

Surface morphology analysis of the samples, annealed at 600 °C, shows fine-grained crystallites. They are often laid out as “islands”, which reflects the microstructure of the Rex 734 substrate. Similar sample surface morphology is observed after annealing at 800 °C. Big “protruding” porous grains are visible on the surface with fine-grained, homogeneously distributed, globular crystallites. Results of EDS analysis (Table 8) show that these grains have higher levels of O and Fe.

**Table 8:** Chemical composition of grains crystallized on the samples annealed at 800 °C

	As prepared (before corrosion)				After anodic polarization			
Element	Point (1)		Point (2)		Point (3)		Point (4)	
	at.%	wt%	at.%	wt%	at.%	wt%	at.%	wt%
O	62.618	32.531	56.990	27.266	64.847	34.672	10.578	3.281
Si	0.059	0.054	0.319	0.268	0.143	0.135	0.911	0.496
Ti	0.132	0.204	0.440	0.629	0.097	0.155	0.192	0.178
Cr	3.760	6.348	11.043	17.170	2.258	3.924	20.083	20.245
Mn	3.533	6.302	1.936	3.180	2.037	3.740	3.558	3.790
Fe	28.163	51.068	23.121	38.611	30.215	56.389	54.883	59.423
Ni	1.570	2.991	4.223	7.412	0.238	0.467	7.789	8.864
Nb	0.166	0.501	0.759	2.109	0.084	0.261	0.139	0.251
Mo	0.000	0.000	1.169	3.355	0.080	0.257	1.867	3.472

In the case of samples with TiO_2_ layers annealed at temperatures of 600 and 800 °C, anodic polarization causes local corrosion damage by partial removal of the TiO_2_ layer and revealing of the microstructure of the substrate. In the case of samples annealed at 600 °C, this damage was less extensive than in the case of samples heat treated at 800 °C. SEM analysis of samples with TiO_2_ layer annealed at 800 °C shows an additional occurrence of intergranular corrosion effects in the area without the layer. The results of EDS analysis show that the elemental composition of this area is similar to the original elemental composition of the investigated alloy. SEM–EDS analysis also indicates that porous grains with higher levels of Fe and O are still present on the surface, even after anodic polarization.

## Discussion

Considering the results of phase analysis, and the thickness and density of investigated TiO_2_ layers, it was possible to describe the type of layer-substrate connection, as well as the structure of the coating, formed at different temperatures.

On the basis of the phase analysis, it was stated that layers formed at 200 and 400 °C had an amorphous structure. Simultaneously, the density values of these layers indicated that crystal forms of titania were also present. The determined density value of the TiO_2_ layer annealed at 200 °C was close to the density value of anatase. This could be explained by the formation of anatase microcrystals in the amorphous structure of titania. Analogous mixed structures have also been reported in literature concerning research into TiO_2_ layers on Ti [38–41]. Increasing the annealing temperature up to 400 °C caused the formation of a thicker and much denser layer than the layer created at a lower temperature. At both described annealing temperatures, the interaction between substrate and deposited TiO_2_ layers probably had an adhesive character.

Phase analyses of TiO_2_ layers formed at 600 and 800 °C showed the diffusion process occurring on the interface of the TiO_2_ layer and the Rex 734 substrate. As a result, new phases were formed on the basis of TiO_2_ and alloying elements. Based on phase analysis and density measurements, it could be stated that samples annealed at 600 °C were covered by the NiTiO_3_ phase rather than pure TiO_2_. This NiTiO_3_ phase appeared on the surface as a result of Ni diffusion from the alloy to the TiO_2_ layer. Analogous measurements for samples annealed at 800 °C showed that coatings formed onto these samples are double layered. The inner layer consisted of a Fe_2_TiO_4_ phase, while the outer layer was an NiTiO_3_ phase. Moreover, a few crystallized, porous grains of Fe_2_O_3_ phase could be observed on the surface. They were also detected in SEM investigations. At both described annealing temperatures, the interaction between substrate and deposited TiO_2_ layers had a diffusive character.

Analyzing the impact of TiO_2_ layers on the corrosion properties of Rex 734 alloy, it should be concluded that these layers annealed at all the temperatures used improved the corrosion resistance of this alloy. The effect of a shifted corrosion potential towards more anodic values, the increase in polarization resistance, reduced porosity, corrosion current and *CR* can be explained by the protective properties of TiO_2_ coating. This protective effect is also evident in the whole passive range for all samples.

An analysis of the potentiodynamic characteristics shows that TiO_2_ layers annealed at 400 °C give the greatest improvement in these properties. The layers annealed at this temperature and at a temperature of 200 °C, both adhesively bound to the substrate, considerably reduce the CR by blocking the transport of metallic ions. Pitting corrosion occurring as a result of anodic polarization of the alloy coated with layers annealed at these temperatures may be associated with those precipitates with higher levels of Nb, Mo and Cr, which were detected on the alloy surface. Around these precipitates, the alloy substrate is deficient in Nb, Mo and Cr elements. Therefore, these sites may be susceptible to corrosive attack. It should be noted that the composition of precipitates, given in Sect. 3.6, may be affected by a significant error that results from the small size of these precipitates. A similar problem with the interference of the surrounding matrix was signalled by Pan et al. [42]. He reported that EDX analysis showed that Z-phase precipitations contain ca. 27 % Cr and ca. 6 % Mo, while typical levels of these elements in the substrate amount to 22 and 3 %, respectively. The composition of the Z-phase precipitates and the mechanism of their formation is described extensively in the literature [1, 43–48]. Pitting corrosion occurs at locations corresponding to these precipitates [42, 43].

The weakest protective properties are exhibited by TiO_2_ layers annealed at the temperatures of 600 and 800 °C. The main reason for this may be the diffuse nature of the bond between the substrate and the coating. Changes in the chemical composition of TiO_2_ coating due to diffusion of Ni and Fe to the coating, as found in the phase analysis, reveal an increase in the corrosion current and a low value of breakdown potential. Rex 734 alloy heat treated at these temperatures does not have the ability to repassivate pits once formed. Evans et al. [26] and Zhu et al. [27] discussed the adverse effect of diffusion layer thus formed, but mainly in terms of photocatalytic properties. Zhu et al. [27] identified diffusion of Fe and forming of an Fe_2_O_3_ interlayer and rhombohedral Fe_2_O_3_ species on SS 304 steel. The analogical formation of the diffusion layer and the diffusion of alloying elements of the investigated Rex 734 into the TiO_2_ film, both at 600 and 800 °C, are certain to have a crucial influence on the corrosion protective properties of TiO_2_.

Robinson and Jack [46] has shown that, in Rex 734, the Z-phase is formed as inter- and intragranular precipitates in the temperature range of 700–1,000 °C. An extensive analysis of the formation of Z-phase precipitation in austenitic stainless steel at high temperatures was presented by Sourmail [47]. Thermal treatment of stainless steel at high temperatures also causes the formation of ageing-induced intergranular precipitates (*χ*-phase) [43]. The presence of *χ*-phase, which normally occurs at grain boundaries, depletes the chromium content leading to intergranular corrosion [49]. The presence of such a phase has proven to be highly sensitive to alloy processing parameters such as the cooling rate after a final heat treatment. The *χ*-phase can be avoided during production by sufficiently rapid cooling [43]; however, in our studies we used the gradual cooling of the samples in the oven, to avoid the shrinkage and cracking of the applied TiO_2_ layers. Under such conditions, formation of Z-phase and *χ*-phase intermetallic precipitates is very possible. The presence of these precipitates would explain intergranular corrosion and the revealing of the microstructure of the substrate due to anodic polarization of Rex 734 alloy samples with TiO_2_ layer annealed at 800 °C. An analogous alloy microstructure was obtained in corrosion studies in 0.5 M H_2_SO_4_ [50].

## Summary

Structural analysis and corrosion tests of titanium dioxide layers deposited onto the surface of ISO 5832-9 biomedical alloy by the sol–gel method and annealed at temperatures in the range of 200–800 °C were performed in this study. The morphology, chemical composition, crystallinity, thickness and density of deposited TiO_2_ layers were determined using suitable electron and X-ray measurement methods. Anticorrosion properties of TiO_2_ layers were studied using electrochemical methods. The results may be summarized as follows:

1. The sol–gel procedure used in this study allows homogenous and crack-free TiO_2_ layers to be obtained on Rex 734 biomedical alloy. The character of interaction between substrate and deposited TiO_2_ layers depends on annealing temperature. At temperatures of 200 and 400 °C, the interaction has an adhesive character, while at 600 and 800 °C it has a diffusive character.
2. The structure of TiO_2_ layers depends on annealing temperature. Below the temperature of 400 °C, TiO_2_ layers are amorphous or amorphous with anatase crystallites. At temperatures higher than 600 °C, new phases containing Ti and alloying elements (Ni and/or Fe) are formed.
3. All the obtained TiO_2_ coatings exhibit anticorrosion properties. Their protective properties are related to the crystalline structure and character of the substrate–layer interaction. The best anticorrosion properties are exhibited by coatings of amorphous structure with anatase microcrystalities and an adhesive connection character with the substrate of the Rex 734 biomedical alloy, whereas the weakest protective properties are noted in diffusive layers, which contain new TiO_2_-based phases instead of pure TiO_2_ crystal structures.
4. During heat treatment of the Rex 734 alloy with TiO_2_ coating, some precipitates are formed. These precipitates may have an impact on the type of corrosion damage created as a result of anodic polarization. At low temperatures (200 and 400 °C) pitting corrosion with circular pits occurs, while at higher temperatures (600 and 800 °C) local corrosion by partial removal of TiO_2_ layer is noted.

In conclusion, from the corrosion point of view the best TiO_2_ sol–gel coatings for stainless steel intended for biomedical applications seem to be those obtained at 400 °C.

## References

[1] Giordani EJ, Guimaraes VA, Pinto TB, Ferreira I (2004). Effect of precipitates on the corrosion–fatigue crack initiation of ISO 5832-9 stainless steel biomaterial. Int J Fatigue.

[2] Uggowitzer PJ, Magdowski R, Speidel MO (1996). Nickel free high nitrogen austenitic steels. ISIJ Int.

[3] Thomman UI, Uggowitzer PJ (2000). Wear–corrosion behavior of biocompatible austenitic stainless steels. Wear.

[4] British Stainless Steel Association. Selection of stainless steels for surgical implants. http://www.bssa.org.uk/topics.php?article=138. Accessed 20 June 2013.

[5] Liu C, Bi Q, Matthews A (2001). EIS comparison on corrosion performance of PVD TiN and CrN coated mild steel in 0.5 N NaCl aqueous solution. Corros Sci.

[6] Battison GA, Gerbasi R, Porchia M (1994). Influence of substrate on structural properties of TiO_2_ thin films obtained via MOCVD. Thin Solid Films.

[7] Fallet M, Mahdjoub H, Gautier B, Bauer JP (2001). Electrochemical behavior of ceramic sol-gel coatings on mild steel. J Non-Cryst Solids.

[8] Pang X, Zhitomirsky I, Niewczas M (2005). Cathodic electrolytic deposition of zirconia films. Surf Coat Technol.

[9] Guillard C, Beaugiraud B, Dutriez C, Herrmann JM, Jaffrezic H, Jaffrezic-Renault N, Lacroix M (2002). Physicochemical properties and photocatalytic activities of TiO_2_-films prepared by sol–gel methods. Appl Catal B.

[10] Velten D, Biehl V, Aubertin F, Valeske B, Possart W, Breme J (2002). Preparation of TiO_2_ layers on cp-Ti and Ti6Al4 V by thermal and anodic oxidation and by sol-gel coating techniques and their characterization. J Biomed Mater Res.

[11] Alam MJ, Cameron DC (2002). Preparation and characterization of TiO_2_ thin films by sol-gel method. J Sol-Gel Sci Technol.

[12] Galliano P, De Damborenea JJ, Pascual MJ, Duran A (1998). Sol-gel coatings on 316L steel for clinical applications. J Sol-Gel Sci Technol.

[13] Wang D, Bierwagen GP (2009). Sol–gel coatings on metals for corrosion protection. Prog Org Coat.

[14] Velten D, Eisenbarth E, Schanne N, Breme J (2004). Biocompatible Nb_2_O_5_ thin films prepared by means of the sol-gel process. J Mater Sci Mater Med.

[15] Tayade RJ, Surolia PK, Kulkarni RG, Jasra RV (2007). Photocatalytic degradation of dyes and organic contaminants in water using nanocrystalline anatase and rutile TiO_2_. Sci Technol Adv Mater.

[16] Kopac T, Bozgeyik K (2010). Effect of surface area enhancement on the adsorption of bovine serum albumin onto titanium dioxide. Coll Surf B.

[17] Głuszek J, Masalski J, Furman P, Nitsch K (1997). Structural and electrochemical examinations of PACVD TiO_2_ films in Ringer solution. Biomaterials.

[18] Shen GX, Chen YC, Lin L, Lin CJ, Scantlebury D (2005). Study on a hydrophobic nano-TiO_2_ coating and its properties for corrosion protection of metals. Electrochim Acta.

[19] Shen GX, Chen YC, Lin CJ (2005). Corrosion protection of 316 L stainless steel by a TiO_2_ nanoparticle coating prepared by sol–gel method. Thin Solid Films.

[20] Shan CX, Hou X, Choy KL (2008). Corrosion resistance of TiO_2_ films grown on stainless steel by atomic layer deposition. Surf Coat Technol.

[21] Atik M, de Lima Neto P, Avaca LA, Aegerter MA (1995). Sol-gel thin films for corrosion protection. Ceram Int.

[22] Nazeri A, Trzaskoma-Paulette PP, Bauer D (1997). Synthesis and properties of cerium and titanium oxide thin coatings for corrosion protection of 304 stainless steel. J Sol-Gel Sci Technol.

[23] Krishna DSR, Sun Y (2005). Thermally oxidised rutile-TiO_2_ coating on stainless steel for tribological properties and corrosion resistance enhancement. Appl Surf Sci.

[24] Balamurugan A, Kannan S, Rajeswari S (2005). Evaluation of TiO_2_ coatings obtained using the sol–gel technique on surgical grade type 316L stainless steel in simulated body fluid. Mater Lett.

[25] Vasconcelos DCL, Nunes EHM, Sabioni ACS, da Costa JCD, Vasconcelos WL (2012). Structural characterization and corrosion behavior of stainless steel coated with sol-gel titania. J Mater Eng Perform.

[26] Evans P, English T, Hammond D, Pemble ME, Sheel DW (2007). The role of SiO_2_ barrier layers in determining the structure and photocatalytic activity of TiO_2_ films deposited on stainless steel. Appl Catal A.

[27] Zhu Y, Zhang L, Wang L, Fu Y, Cao L (2001). The preparation and chemical structure of TiO2 film photocatalysts supported on stainless steel substrates via the sol–gel method. J Mater Chem.

[28] Piwoński I (2007). Preparation method and some tribological properties of porous titanium dioxide layers. Thin Solid Films.

[29] Fritz SE, Martin SM, Frisbie CD, Ward MD, Toney MF (2004). Structural characterization of a pentacene monolayer on an amorphous SiO_2_ substrate with grazing incidence X-ray diffraction. J Am Chem Soc.

[30] Dutta P (2000). Grazing incidence X-Ray diffraction. Curr Sci.

[31] Kogan V, Bethke K, Vries R (2003). Applying X-rays in material analysis. Nucl Instrum Method A.

[32] Stoev KN, Samurai K (1999). Review on grazing incidence X-ray spectrometry and reflectometry. Spectrochimi Acta B.

[33] Kolbe M, Beckhoff B, Krumrey M, Ulm G (2005). Comparison of reference-free X-ray fluorescence analysis and X-ray reflectometry for thickness determination in the nanometer range. Appl Surf Sci.

[34] Tato W, Landolt D (1998). Electrochemical determination of the porosity of single and duplex PVD coatings of titanium and titanium nitride on brass. J Electrochem Soc.

[35] Diaz B, Harkonen E, Światowska J, Maurice V, Seyeux A, Marcus P, Ritala P (2011). Low-temperature atomic layer deposition of Al_2_O_3_ thin coatings for corrosion protection of steel: surface and electrochemical analysis. Corros Sci.

[36] ASTM G 102–89. Standard practice for calculation of corrosion rates and related information from electrochemical measurements. 2004.

[37] Takemoto S, Hattori M, Yoshinari M, Kawada E, Oda Y (2005). Corrosion behavior and surface characterization of titanium in solution containing fluoride and albumin. Biomaterials.

[38] Shibata T, Zhu YC (1995). The effect of film formation conditions on the structure and composition of anodic oxide films on titanium. Corros Sci.

[39] Sul YT, Johansson CB, Jeong Y, Albrektsson T (2001). The electrochemical oxide growth behaviour on titanium in acid and alkaline electrolytes. Med Eng Phys.

[40] Sul YT, Johansson CB, Petronis S, Krozer A, Jeong Y, Wennerberg A, Albrektsson T (2002). Characteristics of the surface oxides on turned and electrochemically oxidized pure titanium implants up to dielectric breakdown: the oxide thickness, micropore configurations, surface roughness, crystal structure and chemical composition. Biomaterials.

[41] Sul YT (2003). The significance of the surface properties of oxidized titanium to the bone response: special emphasis on potential biochemical bonding of oxidized titanium implant. Biomaterials.

[42] Pan J, Karlen C, Ulfvin C (2000). Electrochemical study of resistance to localized corrosion of stainless steels for biomaterial applications. J Electrochem Soc.

[43] Ornhagen C, Nilsson JO, Vannevik H (1996). Characterization of a nitrogen-rich austenitic stainless steel used for osteosynthesis devices. J Biomed Mater Res.

[44] Antunes RA, de Oliveira MCL (2012). Corrosion fatigue of biomedical metallic alloys: mechanisms and mitigation. Acta Biomater.

[45] Erneman J, Schwind M, Liu P, Nilsson J-O, Andren H-O, Agren J (2004). Precipitation reactions caused by nitrogen uptake during service at high temperatures of a niobium stabilised austenitic stainless steel. Acta Mater.

[46] Robinson EW, Jack DH (1985). Precipitation of Z-phase in a high-nitrogen stainless steel. J Heat Treat.

[47] Sourmail T (2001). Precipitation in creep resistant austenitic stainless steels. Mater Sci Technol.

[48] Giordani EJ, Jorge AM, Balancin O (2006). Proportion of recovery and recrystallization during interpass times at high temperatures on a Nb- and N-bearing austenitic stainless steel biomaterial. Scr Mater.

[49] Xu W, San Martin D, Rivera-Díaz-del-Castillo PEJ, van der Zwaag S (2006). Modelling chi-phase precipitation in high molybdenum stainless steels. Adv Mat Res.

[50] Burnat B. Investigation of electrochemical corrosion of FeCr biomedical alloys. PhD Thesis. University of Lodz. 2008.

